# Undergraduate research in medical schools in Pakistan: Relevance, Needs, and Importance

**DOI:** 10.12669/pjms.39.5.7137

**Published:** 2023

**Authors:** Asif Ali Burney, Ikram Ali Burney, Kabir Dherwani

**Affiliations:** 1 Asif Ali. Burney, MBBS, FCPS. Isra University, Hyderabad, Pakistan; 2 Ikram Ali Burney, MBBS, PhD. Sultan Qaboos Comprehensive Cancer Care & Research Center, Muscat 123, Oman.; 3 Kabir Dherwani, MBBS, M.M.Ed. Isra University, Hyderabad, Pakistan

**Keywords:** Medical, Research, Undergraduate, Pakistan, underdeveloped

## Abstract

Recent trends in medical education demand practicing physicians to be competent in playing multiple roles. Life-long learning skills underpinned by research & scholarly activity will enable them to play these roles adequately. Therefore, the introduction of structured training in research during early undergraduate years is pivotal. This would enable trainee physicians to develop essential skills; the institutions will grow in terms of visibility & ranking; the trainers and supervisors will be satisfied in terms of research productivity and, most importantly the patients will benefit in terms of improved clinical outcomes.

Over the past few years, medical practice has changed significantly. An aging population, societal demands, informed patients, increased litigation, and regulatory requirements have all resulted in a drive to produce ‘competent’ physician. A competent physician is defined as a doctor who is not only able to diagnose and manage illness, but can also communicate effectively, exhibit high levels of professionalism, has a good understanding of biomedical scientific principles, is a lifelong learner, and is a scholar.^1^ To produce competent physicians, medical curricula across the world have also undergone a phase of transition. From an apprenticeship-based curriculum, through the discipline-based curriculum, we now live in the era of integrated, problem-based, outcome-based, and competency-based curricula.^2^ Several medical schools have adopted integrated curricula and introduced teaching of research methodology at the undergraduate level.

Needs and requirements aside, there are several benefits of introducing research early in the undergraduate phase for students, the faculty, and the medical school/university. Learning research methodology enable students to develop critical thinking skills such as problem-solving; analyzing information; synthesizing information from different sources, evaluating credibility of sources, assessing strengths or weakness of arguments and developing innovative solutions to complex problems; oral and written communication skills, and project management and evidence-based thinking experience of conducting research.^3^

The faculty members get an opportunity to enhance their research agenda through extra pairs of hands, and this may lead to increased productivity in terms of supervision, research grants, presentations, and publications.^4^ The medical school or the university also benefits from the process; increased numbers of publications and presentations at national and international forums not only enhance the visibility of the university but also result in a higher ranking.^5^

Research methods can be taught both by didactic teaching and by providing experiential learning opportunities.^6^ Learning research methods by carrying out the research work results in both short and long-term advantages for students. In the short term, students get to understand the research process, develop an understanding of how to tackle a scientific problem, learn various types of laboratory techniques, and develop the ability to interpret results and analyze the data. As a result, they are better able to integrate the underpinning theory with practice. Students also get an opportunity to present the data both verbally and in written form, and by publishing, they get an opportunity to enhance their resumes, enabling them to have subsequent placements in post-graduate programs. In the longer term, students learn to work independently, as well as a team-member, define their career path, develop self-confidence, and have the satisfaction that they actually contributed to knowledge Additionally, the experience acquired during the undergraduate years may help the students to build up self-efficacy in research leading to a career in science.^3^

From the student’s perspective, more than 90 percent of students who did research projects during the MBBS program recommended this for their juniors while a similar percentage who did not get the opportunity expressed their interest in doing research.^7^

Whereas students, faculty, and the university enjoy the advantages of undergraduate research, the receivers of the healthcare, the patients, may also benefit. Although it is difficult to generate prospective data to see whether research done in undergraduate years leads to better patient outcomes, it has been shown that the clinical outcomes of patients treated in hospitals that are actively involved in research are better, compared to the outcomes of patients treated in hospitals which do not participate in research activities. For example, the overall survival of newly diagnosed patients with ovarian cancer was superior if the patients were treated in hospitals that were active in research, as compared to the patients who were treated in the ‘service’ hospitals.

Amongst the 165 hospitals in Germany, with an almost equal number of research-active and research-inactive hospitals, the median overall survival of patients treated in research-active hospitals was 35 months, compared to 25 months for patients treated in research-inactive hospitals. Moreover, research-active hospitals were able to provide treatment according to existing clinical practice guidelines, compared to other hospitals.^8^ The (CRUSADE) Can Rapid Stratification of Unstable Angina Patients Suppress Adverse Outcomes with Early Implementation of the American College of Cardiology/American Heart Association Guidelines) study reported better cardiovascular outcomes in items of short-term mortality for patients treated in hospitals that participate in clinical trials.^9^ Hospitals that participated in trials had higher adjusted guideline adherence than non-participating hospitals. It is known that the outcomes of patients participating in clinical trials are better than the outcomes of patient treated in a real-world setting. However, in the examples cited above, patients were not treated in clinical trials. They just happen to have been treated in hospitals active in research of any sort. The question may be asked as to why the outcomes were better in ‘research-active’ hospitals. The triad of structure-process-outcome has been described by Donabedian.^10^ Although the ‘structure’ of research-active and research-inactive hospitals is almost similar, research activity itself significantly changes the ‘process’ of care, leading to improved ‘outcomes’.

The College of Physicians and Surgeons of Pakistan has made study of research methods obligatory for post-graduate examinations. However, at the undergraduate level, teaching research and its methods is sporadic, usually student-driven by motivated medical students looking to enhance their chances of acceptance into post-graduate programs abroad.^11^ At the time of writing, there are 45 medical colleges in the public sector and 72 in the private sector in Pakistan. There is an increasing recognition of modernizing the curricula in both sectors.^12^ It is imperative that structured teaching of research methods be introduced at the undergraduate level. It may be pertinent to keep in mind the challenges students face toward research, such as lack of knowledge, lack of mentoring, and lack of time. Furthermore there are certain hindrances in teaching research methodology at institutional levels, such as, lack of electronic medical records in several hospitals, limited access to database, lack of financial support, and preference of some medical schools for instruction over research.^13^

The importance of including research methodology as a subject in undergraduate medical curricula is increasingly being recognized, and students are aware of the need for research training, and seek to have more opportunities for experiential learning in their curricula. A recently reported cross-sectional study including 500 medical students and 50 research mentors concluded that the research activities were mutually beneficial and supported the idea that other medical schools may also benefit.^14^,^15^

Therefore, we suggest that introduction of undergraduate research programs require careful planning and coordination, starting with the defining the goals and objectives of the program to guide its development. Secondly identification of resources and provision of funding to support the program by the university from academic or donating industries. Thirdly, recruiting faculty members who are committed to mentor undergraduate students and have requisite research experience to support students throughout the process of learning and conducting research. Fourthly, providing training and support to the faculty and students to help them develop skills needed to conduct research such as research methodology courses, workshops on data analysis and communication skills. Moreover, establishing timelines to monitor the progress so as to complete research projects in a timely manner. Lastly, an evaluation mechanism must be established to determine the effectiveness of teaching research and identify areas for improvement by analyzing program outcomes.

## CONCLUSION

In conclusion, the introduction of research methodology and learning opportunities in the undergraduate curriculum not only help to produce competent physicians but also may enhance patient outcomes as the students and their mentors work in research-active environments. The curricula need to be restructured not only to include courses in research methods but also to provide students with opportunities for experiential learning. There is an urgent need for dialogue to incorporate these courses into the curricula in Pakistan that follow a systematic approach as suggested by the authors.

### Authors Contribution:

**AAB**: Conceived, designed, & did editing of manuscript.

**IAB**: Review, designed and manuscript writing.

**KD**: Did literature review and edit manuscript and responsible for the accuracy of the work.
